# Nuclear Condensates: New Targets to Combat Parasite Immune Evasion? 

**DOI:** 10.3389/fcimb.2022.942200

**Published:** 2022-07-12

**Authors:** Vanessa Luzak

**Affiliations:** ^1^ Department of Physiological Chemistry, Biomedical Center, Ludwig-Maximilians-Universität München, Munich, Germany; ^2^ Institute of Experimental Parasitology, Department of Veterinary Sciences, Ludwig-Maximilians-Universität München, Munich, Germany

**Keywords:** antigenic variation, gene expression, nuclear organization, membrane-less bodies, trypanosomes

## Introduction

Antigenic variation is a successful strategy employed by numerous pathogens to evade the host immune system. It describes the frequent exchange of antigens, which prevents detection and elimination by the host immune system. Strikingly, pathogens from diverse phyla undergo antigenic variation, thereby underlining the efficiency of this strategy for pathogen survival (Deitsch et al., 2009). While the underlying molecular mechanisms are most likely different for each pathogen species, identifying the common principles of antigenic variation may open up new avenues to combat infectious diseases.

African trypanosomes are commonly used model organisms to study antigenic variation due to their large antigen repertoire of more than 2000 antigen-coding genes (Horn, 2014) as well as their great compatibility with different experimental setups. It is important to note that trypanosomes are evolutionarily distant from other commonly used eukaryotic model organisms and exhibit a particular organization of transcription: Protein-coding genes are organized in long polycistronic transcription units (PTUs), each containing hundreds of genes. Transcription initiation occurs in a rather unregulated manner at transcription start sites of those PTUs and pre-mRNA maturation is mediated by *trans*—splicing of a common spliced leader RNA to the 5´end of each mRNA. Due to the unregulated nature of transcription, transcript levels are mainly regulated on the post-transcriptional level in the parasite.

To establish long-lasting infections in the mammalian blood stream, two requirements have to be met: (1) each trypanosome selectively expresses one antigen and only few different antigens are expressed in the population at any time; (2) the expressed antigen is frequently exchanged. Notably, antigen expression is governed in a specific manner which differs from other protein-coding genes in the parasite: An antigen-coding gene can only be expressed when located in one of fifteen polycistronic expression sites (ESs), of which only one is active in each cell. To ensure stable levels of antigen mRNA, the active ES is expressed by highly processive RNA polymerase I (Pol I) within a nuclear body referred to as expression site body (ESB) (Navarro and Gull, 2001). *Trans*-splicing ensures maturation of the polycistronic pre-mRNA and mature antigen mRNA is additionally stabilized by m^6^A RNA modifications in the poly-A tail (Viegas et al., 2022). Taken together, trypanosomes have evolved an entire ensemble of molecular mechanisms that ensure extremely high levels of antigen mRNA, organized in a multi-step expression process. However, it has remained elusive how such multi-step expression process is coordinated exclusively at one antigen expression sites, while the other 14 expression sites remain silent.

**Figure 1:**
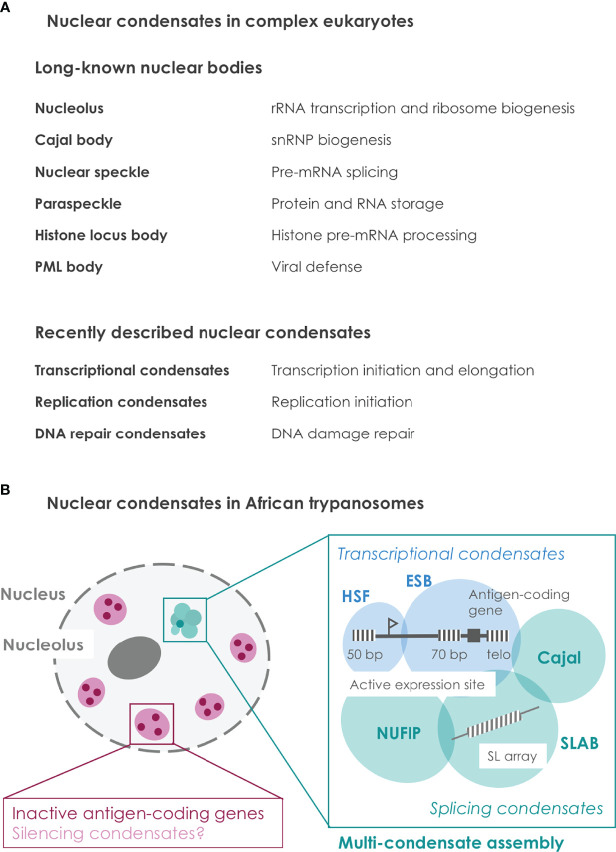
Nuclear condensates in complex eukaryotes and in African trypanosomes. **(A)** Overview of nuclear condensates characterized in complex eukaryotes. While some nuclear bodies are stable during G1 phase and have been described decades ago, other condensates form more dynamically and have been described only recently. A brief description of the respective condensate function is provided (Spegg and Altmeyer, 2021). **(B)** Illustration of so far known nuclear condensates in African trypanosomes. The active antigen gene is embedded within a multi-condensate assembly (Faria et al., 2021; Budzak et al., 2022). Spatial integration of transcription and splicing condensates at the active antigen gene presumably coordinates the multi-step process of antigen expression. In contrast, inactive antigen genes are located away from the multi-condensate assembly. Transcriptional silencing of antigen genes might be mediated by condensate formation. Repetitive sequences are illustrated as striped boxes. ESB, expression site body; HSF, highly SUMOylated focus; SLAB, spliced leader array body; Cajal, Cajal body; NUFIP, NUFIP body; SL, Spliced leader; 50 bp, 50 bp repeats; 70 bp, 70 bp repeats; telo, telomeres.

Nuclear condensates define specific functional compartments within the nucleoplasm and contain different macromolecules such as DNA, RNA and proteins (Bhat et al., 2021) (**Figure 1A**). Lacking a lipid membrane, condensates can assemble and disassemble in a dynamic manner, thereby creating specific environments on demand. Proteins involved in condensate formation often harbor two different types of domains: ordered and intrinsically disordered domains. Ordered domains with a defined structure engage in strong interactions with a certain stoichiometry, whereas intrinsically disordered domains (IDRs) lack any defined structure and form multivalent interactions with other macromolecules. Such weak multivalent interactions allow supra-stochiometric recruitment of macromolecules and can thereby enhance biological reactions (Guo et al., 2019; Laflamme and Mekhail, 2020). Further, multivalent interactions can enable the formation of biological condensates, e.g. *via* a process referred to as liquid-liquid phase separation, and thereby create subcellular environments with specific functions (Bhat et al., 2021). Posttranslational protein modifications (PTMs) as well as physical parameters such as temperature or pH, modulate the strength of multivalent interactions and condensate formation. Taken together, condensates have been observed in various biological processes in diverse species and we only begin to understand their regulatory potential (Banani et al., 2017; Sabari et al., 2020; Lyon et al., 2021)

Interestingly, recent studies in trypanosomes revealed that an entire ensemble of nuclear condensates plays a crucial role during parasite immune evasion. Namely, the active antigen-coding gene was found embedded within a nuclear multi-condensate assembly (Faria et al., 2021; Budzak et al., 2022)(**Figure 1B**). This finding provides an attractive model to explain (1) how one antigen-coding gene is exclusively chosen to be activated and (2) how the multi-step process of highly efficient antigen expression is spatially coordinated.

## Transcription and Splicing Condensates in Eukaryotic Gene Expression

The first nuclear condensate involved in trypanosome antigen expression was described two decades ago as an extranucleolar Pol I body associated with the actively expressed antigen-coding gene and is commonly referred to as expression site body (ESB) (Navarro and Gull, 2001). More recently, the active antigen-coding gene was shown to interact with the SL-RNA array, a genomic locus involved in mRNA maturation by encoding the essential *trans*-splicing substrate (Faria et al., 2021), as well as with the respective spliced leader array body (SLAB) comprised of different splicing-related proteins (Budzak et al., 2022). Surprisingly, even more nuclear condensates were found to be associated: also the Cajal body, a highly SUMOylated condensate and the newly identified NUFIP body are located adjacent to the active antigen-coding gene (Budzak et al., 2022). Taken together, these findings demonstrate that the active antigen-coding gene is not only associated with a single nuclear condensate such as the ESB, but that it is embedded in the context of multiple condensates, which can be referred to as a multi-condensate assembly (**Figure 1B**).

The specific function of each condensate in this assembly remains to be experimentally characterized. However, the molecular composition of two condensate groups allows speculation about their function: (1) the ESB and the highly SUMOylated condensate contain factors involved in antigen transcription (potential transcriptional condensates) (Navarro and Gull, 2001; Saura et al., 2019); (2) the SLAB, the NUFIP and the Cajal body contain factors involved in mRNA processing (potential splicing condensates) (Faria et al., 2021; Budzak et al., 2022). In other eukaryotes, the formation of transcription and splicing condensates is a transient process, with a life time of 5 to 11 seconds for transcriptional condensates (Cisse et al., 2013; Cho et al., 2016), that is coordinated by phosphorylation of the C-terminal domain (CTD) of Pol II (Guo et al., 2019; Bhat et al., 2021). In trypanosomes, the active antigen is transcribed by Pol I, which does not – to our knowledge – harbor a comparable CTD that could coordinate co-transcriptional condensate formation. Thus, arranging both antigen transcription condensates in close proximity to three splicing condensates might reflect a CTD-independent mechanism to coordinate both processes co-transcriptionally.

In contrast to the transient transcription and splicing condensates that form co-transcriptionally in other eukaryotes, the multi-condensate assembly in trypanosomes seems rather stable according to imaging data from fixed cells (Faria et al., 2021; Budzak et al., 2022). Therefore, a comparison to stable condensates from other eukaryotes is appropriate, such as super-enhancers or nuclear speckles, which mediate robustness in the otherwise stochastic gene expression process. As an example, super-enhancers are encoded by several sequence elements, which serve as recruitment platforms to form stable transcription-related condensates with a life time of several minutes or even longer (Cho et al., 2018; Sabari et al., 2018). Mainly lineage-specific genes are selectively regulated by the interaction with super-enhancers, which require robust transcription while cell identity is formed and maintained (Hnisz et al., 2013; Monahan et al., 2019). As a second example, nuclear speckles are stable condensates that contain many splicing-related factors; they remain intact during interphase and are only disassembled during cell division (Galganski et al., 2017). Highly transcribed house-keeping genes are located close to nuclear speckles in different cell types (Galganski et al., 2017; Quinodoz et al., 2018; Zhang et al., 2020) and heat shock genes were observed to move towards nuclear speckles upon activation (Kim et al., 2020). Thus, it was suggested that nuclear speckles act as stable splicing condensates mediating robust expression of genes located in spatial proximity.

Collectively, the comparison of antigen transcription and splicing condensates in trypanosomes to the respective condensates from other eukaryotes allows a careful first interpretation: the stable association of several nuclear condensates with specific functions at the active antigen-coding gene might spatially coordinate the multi-step-process of antigen expression and confer robustness to it. The ESB Pol I reservoir and the highly SUMOylated body most probably mediate high and robust antigen transcription rates. Further, the three splicing condensates, SLAB, NUFIP and Cajal body, fulfill the high demand for efficient antigen mRNA processing, which is required for successful antigen expression. It remains to be shown which specific function each condensate possesses and which additional processes, such as e.g. m^6^A modification, are coordinated within the multi-condensate assembly.

## Potential Condensate Seed Factors Involved in Parasite Immune Evasion

Seeding of nuclear condensates is thought to be mediated by spatially restrained chromatin elements such as DNA, histones or RNA, which recruit freely diffusible proteins to a given locus (Bhat et al., 2021). Repetitive DNA sequences contain a multitude of potential binding sites and can thereby serve as especially efficient recruitment platforms, as well as the arising repetitive transcripts (Frank and Rippe, 2020; Bhat et al., 2021).

Interestingly, several repetitive DNA sequences are implicated in antigen expression in trypanosomes, indicating a potential role as seed structures (**Figure 1B**). On the one hand, each antigen expression site harbors three repetitive DNA elements: (1) 50 bp repeats are located upstream of the expression site promoter; (2) 70 bp repeats are located upstream of the antigen-coding gene at the end of each expression site; (3) telomeric repeats start downstream of the antigen coding gene (Hertz-Fowler et al., 2008). The role of the trypanosome-specific 50 bp and 70 bp repeats has remained unclear, though their presence in each of the 15 antigen expression sites suggests an important regulatory role. Notably, nuclear condensates can not only enhance biological reactions but also fulfill an inhibitory or silencing function by excluding specific molecules from a certain nuclear region. In this context, the 50 bp, 70 bp or telomeric repeats could serve as condensate seed structures that establish stable silencing of inactive expression sites. Alternatively, transcripts from the 70 bp repeats, which are transcribed in the active expression site, could play a role during the multi-condensate assembly to ensure robust expression of this particular antigen expression site. On the other hand, the SL-RNA array is a repetitive genomic locus (Sutton and Boothroyd, 1986) and is part of the splicing-related SLAB condensate in the multi-condensate assembly (Budzak et al., 2022). High levels of SL-RNA transcripts most probably recruit splicing factors to this condensate and thereby create an active splicing condensate.

RNA plays a crucial role in condensate formation (Garcia-Jove Navarro et al., 2019; Quinodoz et al., 2021; Bhat et al., 2021) and it remains to be shown which specific role RNA plays for the multi-condensate formation in trypanosomes. Interestingly, experimental evidence suggests that both RNA-related processes, expression site transcription and *trans*-splicing, are required for intact multi-condensate formation. Inhibition of Pol I transcription caused VEX1 and VEX2, two proteins that are part of the multi-condensate assembly, to distribute within the nucleus (Faria et al., 2019). Further, inhibition of *trans*-splicing caused a disruption of the ESB-SLAB assembly (Faria et al., 2021) and resulted in the inhibition of expression site transcription by Pol I (Budzak et al., 2022). Taken together, these experiments suggest that formation of the multi-condensate is functionally linked to the processes of efficient expression site transcription and antigen mRNA processing. In addition, m^6^A RNA modifications were recently detected in the Poly-A tail of the active antigen transcript (Viegas et al., 2022), which are responsible for increased antigen mRNA stability. m^6^A modifications have been implicated in condensate formation (Ries et al., 2019) and it remains to be shown if such RNA modifications play a role in the multi-condensate formation process.

Silencing nuclear condensates are for example observed as heterochromatin condensates in different eukaryotes, which exclude the transcription machinery and thereby ensure transcriptional silencing of genomic sites within the condensate (Bhat et al., 2021). In this context, the kinetoplastid-specific DNA modification Base J could play a role as silencing condensate seed structure, since it is enriched at inactive antigen expression sites (van Leeuwen et al., 1997). Further, two histone variants, H3.V and H4.V, are enriched at inactive antigen expression sites and upon deletion of both variants, silent antigen expression sites are activated (Müller et al., 2018). However, it remains to be shown if such activation is the consequence of disrupting a silencing condensate.

Taken together, there are several potential seed structures for nuclear condensates involved in immune evasion of trypanosomes – some might contribute to condensates involved in active antigen expression, whereas others might regulate condensate-mediated silencing of the remaining antigen expression sites. One possible way to determine which of these factors are indeed capable of inducing condensate formation, is the performance of *in vitro* experiments, as done for other organisms (Alberti et al., 2019; Zamudio et al., 2019). Trypanosome nuclear extracts could be used, which are depleted of endogenous DNA and/or RNA, and *in vitro* generated and fluorescently labeled seed structure molecules could be added - such as repeat-encoding or BaseJ-modified DNA fragments and *in vitro* transcribed RNAs. Condensate formation can be observed under a microscope and the fluorescent label would verify that the seed structure is a component of the formed condensates.

## Potential Regulatory Components of Nuclear Condensates in Trypanosomes

Numerous proteins are enriched in the nuclear condensates around the active antigen-coding gene (Budzak et al., 2022). As a first example of a protein with a specific function in trypanosome condensate biology, the RNA helicase VEX2 was shown to actively exclude all but one antigen expression site from interacting with the SLAB splicing body (Faria et al., 2021). Upon VEX2 depletion, several silent antigen expression sites started to interact with the SLAB body and were activated. Future studies will decipher the specific role of other condensate proteins in selective antigen expression.

Of all protein PTMs, SUMOylation seems to be the most prominent modification with a role in selective antigen expression, since a highly SUMOylated body is located close to the active antigen-coding gene (López-Farfán et al., 2014; Budzak et al., 2022). Previous studies have shown that TbSIZ1/PIAS1 is the SUMO E3 ligase that is responsible for the strong SUMOylation signal and that SUMOylation is required for high antigen expression levels (López-Farfán et al., 2014; Saura et al., 2019). Interestingly, the largest subunit of Pol I is one of the SUMOylated proteins. A study in yeast has shown that, in the context of rDNA repair, SUMOylation serves as a signal to exclude modified proteins from the nucleolar condensate (Capella et al., 2021). The formation of an additional extranucleolar Pol I focus in trypanosomes is a highly unusual phenomenon, since Pol I is usually exclusively localized in nucleoli. Thus, SUMOylation of the largest Pol I subunit in trypanosomes might be critical for Pol I release from the nucleolus and subsequent ESB formation.

Another means of condensate regulation can arise from physical parameters such as temperature. In this context, it is a striking observation that expression site transcription is activated upon a temperature shift from 28°C to 37°C (Kolev et al., 2018). Such temperature shift mimics the conditions when the parasite enters the mammalian blood stream and therefore the time point when antigen expression becomes critical for parasite survival. It is an intriguing possibility that the multi-condensate assembly is temperature sensitive and therefore is induced at 37°C when the parasite enters the host bloodstream. Further, it remains to be shown if changes to higher temperatures than 37°C, such as a fever in the host organism during infection, influence parasite antigen expression or antigen switching.

## Nuclear Condensates as Potential Targets to Manipulate Parasite Immune Evasion

The different factors discussed above with a potential role in trypanosome condensate formation are well-known in the field of parasite immune evasion. It is not the aim of this article to identify previously unknown regulators, but rather to allow the reader to think about known factors in a different context – in the context of condensate biology. The multi-condensate assembly around the active antigen-coding gene in trypanosomes seems critical for selective antigen expression and therefore for successful immune evasion and infection establishment. When selective antigen expression is lost and several antigens are expressed in individual trypanosomes, the infection can be cleared by the immune system quickly (Aresta-Branco et al., 2019). Therefore, developing strategies to selectively dissolve parasite-specific condensates, as it is currently done in the context of cancer or neurodegenerative diseases (Dolgin, 2021), could potentially open up new avenues to interfere with parasite immune evasion.

Antigenic variation mechanisms might vary between different pathogens, but it is likely that condensates are commonly involved in such highly regulated processes; therefore, targeting nuclear condensates involved in antigenic variation could become a universal strategy to combat infectious diseases.

## Author Contributions

The author confirms being the sole contributor of this work and has approved it for publication.

## Funding

VL is funded by the German Academic Scholarship Foundation and by the ERC Starting Grant (3D_Tryps 715466) awarded to Prof. Nicolai Siegel.

## Conflict of Interest

The author declares that the research was conducted in the absence of any commercial or financial relationships that could be construed as a potential conflict of interest.

## Publisher’s Note

All claims expressed in this article are solely those of the authors and do not necessarily represent those of their affiliated organizations, or those of the publisher, the editors and the reviewers. Any product that may be evaluated in this article, or claim that may be made by its manufacturer, is not guaranteed or endorsed by the publisher.
